# An Ultrasensitive High Throughput Screen for DNA Methyltransferase 1-Targeted Molecular Probes

**DOI:** 10.1371/journal.pone.0078752

**Published:** 2013-11-13

**Authors:** Rebecca L. Fagan, Meng Wu, Frédéric Chédin, Charles Brenner

**Affiliations:** 1 Department of Biochemistry, Carver College of Medicine, University of Iowa, Iowa City, Iowa, United States of America; 2 Department of Molecular and Cellular Biology and Genome Center, University of California Davis, Davis, California, United States of America; Albert-Ludwigs-University, Germany

## Abstract

DNA methyltransferase 1 (DNMT1) is the enzyme most responsible for epigenetic modification of human DNA and the intended target of approved cancer drugs such as 5-aza-cytidine and 5-aza-2′-deoxycytidine. 5-aza nucleosides have complex mechanisms of action that require incorporation into DNA, and covalent trapping and proteolysis of DNMT isozymes. Direct DNMT inhibitors are needed to refine understanding of the role of specific DNMT isozymes in cancer etiology and, potentially, to improve cancer prevention and treatment. Here, we developed a high throughput pipeline for identification of direct DNMT1 inhibitors. The components of this screen include an activated form of DNMT1, a restriction enzyme-coupled fluorigenic assay performed in 384 well plates with a z-factor of 0.66, a counter screen against the restriction enzyme, a screen to eliminate DNA intercalators, and a differential scanning fluorimetry assay to validate direct binders. Using the Microsource Spectrum collection of 2320 compounds, this screen identified nine compounds with dose responses ranging from 300 nM to 11 µM, representing at least two different pharmacophores with DNMT1 inhibitory activity. Seven of nine inhibitors identified exhibited two to four-fold selectivity for DNMT1 versus DNMT3A.

## Introduction

In eukaryotes, the most common DNA modification is methylation of the 5 carbon of cytosines, predominately in CpG dinucleotides. Methylation patterns are established and maintained by a family of enzymes known as DNA methyltransferases (DNMTs). *De novo* methyltransferases, DNMT3A and DNMT3B, establish methylation patterns during germ cell and embryonic development. These proteins are aided by DNMT3L, a catalytically inactive isoform that forms complexes with DNMT3A and DNMT3B [1]. Methylation patterns are primarily maintained by DNMT1, which is the most abundant DNMT and possesses specificity for methylation of hemimethylated DNA [2], [3]. DNA methylation is an important epigenetic mark associated with gene repression that plays a critical role in development and differentiation, genome stability, genomic imprinting, X-chromosome inactivation and silencing of retrotransposons [4]. Aberrant DNA methylation has been linked to several diseases including schizophrenia [5], Rett Syndrome [6], autoimmune diseases [6]–[8], hereditary sensory neuropathy, dementia and hearing loss [9], and cancer [10], [11]. In malignancies, normal methylation patterns are disrupted such that global cytosine DNA methylation is reduced, while the regulatory regions of many tumor suppression genes are hypermethylated, resulting in gene silencing [12]. Though genetic changes associated with cancer cannot be corrected, epigenetic changes, such as DNA methylation, are dynamic and amenable to reversal. Epigenetic reprogramming, accomplished by pharmacological targeting of DNMTs, could be expected to result in restoration of a more differentiated and less proliferative state, and regression to a lower degree of drug resistance [13].

The link between the DNMT isozyme DNMT1 and cancer initiation and progression is well established. DNMT1 activity is increased in a variety of malignancies. Several common oncogenic pathways result in the overexpression of DNMT1, either via transcriptional or post-translational mechanisms [14]–[17] and targeting the DNMT1 isozyme for cancer therapy has been validated genetically. For example, lowering the level of DNMT1 with a *Dnmt1* null over *Dnmt1* reduced activity genotype protects against tumor formation in *Apc^(Min)^* mice [18]. In addition, knocking down *DNMT1* with antisense oligonucleotides inhibits neoplasia in cell culture and in mouse tumor models [19], [20]. Though genetic experiments can easily target specific DNMT isozymes, this has not been accomplished by pharmacological agents. Discovery of DNMT1 isozyme specific inhibitors could be of great importance as DNMT3A is inactivated in a high proportion of malignancies such as acute myeloid leukemia [21].

Two distinct classes of demethylating agents have been reported. Nucleoside inhibitors such as 5-aza-cytidine and 5-aza-2′-deoxycytidine are FDA-approved prodrugs for treatment of myelodysplastic syndrome [22]. However, these compounds have complicated mechanisms of action that require their incorporation into DNA. Once incorporated, 5-aza nucleotides act as suicide inhibitors, which trap DNMT isozymes in covalent DNA-protein complexes that are cleared by proteolysis and DNA repair, which contributes to the mechanism of action. 5-aza nucleosides are incorporated nonspecifically into the genome, *i.e.*, not specifically at CpG dinucleotides. Because 5-aza-cytidine metabolism includes formation of 5-aza-CTP and incorporation into RNA, significant toxicity can occur [22], [23]. The second class of reported demethylating agents are non-nucleoside inhibitors. This class contains compounds of broad chemical diversity, many of which have other known targets [24]. These agents include compounds that directly inhibit all DNMT isozymes, such as the SAM-competitive inhibitor SGI-1027 [25], and other compounds lacking in direct experimental evidence for a mechanism of inhibition. The cytotoxicity and lack of specificity of known DNA demethylating agents suggests a need for new DNMT inhibitors. Thus, we set out to discover novel direct inhibitors of DNMT1 enzyme activity.

Two factors that have delayed discovery of DNMT1 enzyme inhibitors are the intrinsic low activity of the enzyme and the laborious nature of traditional DNA methylation assays utilized to examine DNMT activity [26]. We recently solved both problems by discovering that the replication foci targeting sequence (RFTS) domain is an intrinsic, DNA-competitive inhibitor of DNMT1 enzyme activity and by employing a fluorigenic assay with outstanding signal-to-noise [27]. Two other high throughput screening (HTS)-compatible assays have been recently reported [28], [29]. Both assays were used to screen for inhibitors of the *de novo* methyltransferase DNMT3A. The scintillation proximity assay was also used to screen DNMT3A hits against DNMT1 [29]. Here, we report optimization of an endonuclease-coupled DNMT1 assay to screen a 2320 compound library for small molecules that inhibit DNMT1 enzyme activity. Following validation of initial HTS hits, candidate inhibitors were screened for direct binding of DNMT1 in the absence of substrates using differential scanning fluorimetry (DSF). The pipeline described here resulted in discovery of nine previously unreported, direct DNMT1 inhibitors without activity as DNA intercalators. Seven of nine compounds exhibit modest selectivity for DNMT1 versus inhibition of DNMT3A/DNMT3L.

## Materials and Methods

### DNMT Expression and Purification

Truncated forms of human DNMT1 (RFTS-lacking DNMT1, amino acids 621–1616 and RFTS-containing DNMT1, amino acids 351–1616) were expressed and purified as previously reported [27]. Full-length human DNMT3L was expressed and purified as previously described [30]. The catalytic domain of human DNMT3A (CD-DNMT3A; amino acids 611–912) was expressed as an N-terminally his-tagged protein in Rosetta 2(DE3)pLysS competent cells (Novagen). Cells were grown to an optical density of ∼0.5 and then cooled to 18°C. Protein expression was induced with 0.5 mM IPTG and cultures were grown at 18°C for 16 hours. Following cell lysis, protein was purified via metal affinity using Ni-NTA resin (GE Life Sciences). Bound protein was eluted with 50 mM HEPES pH 8, 300 mM NaCl, 400 mM imidazole, 4 mM β-mercaptoethanol, 5% glycerol. Eluted protein was buffer exchanged into 20 mM HEPES pH 8, 0.2 M NaCl, 2 mM DTT, 5% glycerol and further purified using a Heparin HP Hi-Trap column (GE Life Sciences). Bound protein was eluted using a linear gradient from 0.2 to 1.25 M NaCl. Eluted CD-DNMT3A was concentrated and stored at −80°C in 50% glycerol. The CD-DNMT3A•DNMT3L complex was prepared by overnight 1∶1 molar incubation at 4°C in 10 mM Tris pH 7.5, 300 mM NaCl, 2 mM DTT. All proteins were quantified using A_280_ and calculated extinction coefficients.

### Endonuclease-Coupled DNA Methylation Assay

DNMT activity was measured at 37°C using a fluorogenic DNA methylation assay [27]. In short, a hemi-methylated hairpin oligonucleotide with a 5′ fluorophore and a 3′ quencher is methylated by addition of a DNMT and the methyl-donating co-factor, *S*-adenosyl methionine (SAM, HPLC-purified from Sigma). The fully methylated product is a substrate for the restriction endonuclease GlaI (Sibenzyme), which cleaves the oligonucleotide, releasing the fluorophore from the quencher and generating fluorescence. The oligonucleotide substrate (5′-FAM–CCTATGCGmCATCAGTTTTCTGATGmCGmCATAGG-3′-Iowa Black, in which mC denotes 5-methyldeoxycytidylate residues), termed 8006, was synthesized by Integrated DNA Technologies, Coralville, IA. 96 well assays were performed in Co-Star black half-area plates and read in a Biotek Synergy Neo plate reader. 384 well assays were performed in Nunc flat-bottomed black plates and read in a PerkinElmer EnVision plate reader. FAM fluorescence was measured using excitation and emission wavelengths of 485 nm and 520 nm, respectively.

### High-Throughput Assay and Hit Validation

HTS against the Spectrum compound collection (Microsource, Gaylordsville, CT) was performed in eight 384 well plates. Using a Multiflo dispenser (Biotek), 75 µl of assay buffer (10.8 mM Tris-HCl pH 7.5, 1.08 mM MgCl_2_, 1.08 mM DTT, 108 mM potassium glutamate, 0.108 mg/ml BSA, 10.8 µM SAM, 21.6 nM oligonucleotide 8006, 5.4% glycerol) was dispensed into each well. 1 µl of each compound at 880 µM in DMSO (columns 3–22) or DMSO (columns 1, 2, 23 and 24) was added to assay plates using a Microlab Star liquid handling robot (Hamilton) and the plate was incubated at 37°C for 10 minutes. Following this warming step, 5 µl enzyme solution (either a DNMT1-containing solution of 20 mM Tris-HCl, pH 7.5, 300 mM NaCl, 2 mM DTT, 49 nM DNMT1 (amino acids 621–1616), 0.08 U/µl GlaI, 30% glycerol to columns 2–23 or a GlaI control solution of 20 mM Tris-HCl, pH 7.5, 300 mM NaCl, 2 mM DTT, 0.08 U/µl GlaI, 30% glycerol to columns 1 and 24) was added and the plate was again incubated at 37°C for 25 minutes. The final composition of the HTS assay was 11.2 mM Tris-HCl, pH 7.5, 1 mM MgCl_2_, 1.12 mM DTT, 100 mM potassium glutamate, 18.5 mM NaCl, 0.1 mg/ml BSA, 10 µM SAM, 20 nM oligonucleotide 8006, 6.85% glycerol, 0.4 U GlaI, 3 nM DNMT1, 10.9 µM test compound, 1.23% DMSO. Each 384 well plate contained two columns of negative (n) and positive (p) controls for inhibitor activity. Columns 2 and 23 were the n controls with DMSO without test compound. Columns 1 and 24 were the p controls without DNMT1. Thus, 320 compounds were assayed per 384 well plate in columns 3–22.

Assay performance was assessed across the screen using the following parameters: the signal-to-noise (S/N) ratio = (µ_n_−µ_p_)/SD_n_, the signal-to-background (S/B) ratio = µ_n_/µ_p_, and the Z’-factor = 1− 3*(SD_p_ +3SD_n_)/(µ_n_−µ_p_), in which SD_p_ and SD_n_ are standard deviations, and µ_n_ and µ_p_ are means of the n and p control wells of each plate [31].

Data across the screen were normalized to the p (0% DNMT1 activity) and n (100% DNMT1 activity) controls on each plate. Compounds that resulted in at least a 5 SD reduction in observed DNMT1 activity (<41%) were considered potential hits and were re-examined. Validation assays (81 µl total volume, identical buffer conditions to those used in the HTS screen) were performed in triplicate in 96 well plates with FAM fluorescence measured over the course of 1 hour. A control containing GlaI in the absence of DNMT1 was subtracted from each assay condition. Corrected assay traces were plotted and compared to DMSO-containing control traces in Prism (GraphPad Software, Inc).

### Differential Scanning Fluorimetry (DSF) Assay

DSF [32] was used to assess the ability of the validated inhibitors to bind directly to DNMT1 in the absence of DNA and SAM and alter the observed melting temperature (*T_m_*). Assays (25 µl) were conducted in triplicate in a Bio-Rad C1000 Thermal Cycler – CFX Real-Time System using the FRET channel and contained 50 mM HEPES pH 7.5, 150 mM NaCl, 2 µM DNMT1 (amino acids 621–1616), 5X Sypro Orange (Invitrogen Molecular Probes), 100 µM compound, and 1% DMSO. A DMSO control assay in the absence of compounds was also examined. Temperature was increased from 25 to 95°C by 0.5°C per minute. Fluorescence traces were exported and analyzed by fitting to the Boltzmann equation in Prism to determine the *T_m_*.

### GlaI Counterscreen

GlaI inhibitors were identified and excluded using a fluorescence-based assay. Duplicate assays (80 µl) were conducted in 96 well plates. Assays contained 10 mM Tris-HCl pH 7.5, 100 mM potassium glutamate, 1 mM MgCl_2_, 1 mM DTT, 0.1 mg/mL BSA, 5% glycerol, 5 nM oligonucleotide 8007 (5′-FAM-CCTATGmCGmCATCAGTTTTCTGATGmCGmCATAGG-3′-Iowa Black, where mC denotes 5-methyldeoxycytidylate residues, Integrated DNA Technologies, Coralville, IA), 0.2 U GlaI, 11 µM test compound and 1.2% DMSO. Reactions were initiated by the addition of GlaI. Cleavage of the oligonucleotide releases the 5′ FAM fluorophore from the 3′ Iowa Black quencher and generates fluorescence. A control reaction in the absence of GlaI was subtracted from each assay condition to account for background fluorescence of the internally quenched substrate. Corrected time courses were fitted in Prism.

### Detergent Test

The effect of detergent on observed DNMT1 inhibition was examined using the endonuclease-coupled DNA methylation assay. Triplicate assays (81 µl) containing 10 µM SAM, 20 nM oligonucleotide 8006, 0.4 U GlaI, 2 nM DNMT1 (amino acids 621–1616), 5 µM inhibitor and 1.21% DMSO in the presence and absence of 0.01% Triton X-100 were conducted in 96 well plates in a BioTek Neo plate reader at 37°C. A control containing GlaI in the absence of DNMT1 was subtracted from each assay condition. Corrected assay traces were fitted in Prism.

### DNA Intercalation Assay

DNA intercalation was assessed by examining the ability of the compounds to displace ethidium bromide from calf thymus DNA (ctDNA; Sigma). Duplicate assays (100 µl) were conducted in black 96 well plates and contained 15 µg/ml ctDNA, 1.5 µM ethidium bromide and 10 µM compound in methylation assay buffer. DMSO was used as a negative control and daunorubicin (Sigma) was used as a positive control. Fluorescence was measured using excitation and emission wavelengths of 320 nm and 600 nm, respectively.

### Concentration-Dependence Experiments

IC_50_ values for each validated direct inhibitor were determined under identical assay conditions (10 µM SAM and 20 nM oligonucleotide 8006) using the endonuclease-coupled DNA methylation assay in triplicate (80 µl) in a 96 well format. Assays contained 0.8 U GlaI, 2 nM DNMT1 (amino acids 621–1616) and 0.94% DMSO with inhibitor concentration varied from 0 to 10 µM. FAM fluorescence was measured in a Biotek Neo plate reader over the course of 45 minutes. A control containing GlaI in the absence of DNMT1 was subtracted from each assay condition. Corrected assay traces were fitted in Prism and percent activity was determined by comparing to a DMSO containing control. IC_50_ values were determined by fitting the percent activity data using a unity Hill slope in Prism.

### Compound Selectivity

After identifying direct inhibitors of DNMT1 lacking the RFTS domain, inhibitor selectivity was assessed by examining inhibition of RFTS-containing DNMT1 (amino acids 351–1616), CD-DNMT3A/DNMT3L complex, and the bacterial cytosine methyltransferase from M. SssI (New England Biolabs) using the endonuclease-coupled DNA methylation assay. Triplicate assays (100 µl) containing 0.25 mM SAM, 0.2 µM oligonucleotide 8006, 0.8 U GlaI, 20 nM methyltransferase, 20 µM inhibitor and 1% DMSO were conducted in 96 well plates. Following enzyme addition, assay plates were incubated at 37°C for 75 min and fluorescence was measured in a BioTek Neo plate reader. A control containing only GlaI was subtracted from each assay. SGI-1027, a non-selective DNMT inhibitor [25], was used as a positive control and 5-aza-cytidine (Sigma) was used as a negative control. Percent activity was determined by comparing product formation to a DMSO containing control assay.

## Results

### High Throughput Screening

Targeting epigenetic changes is a promising cancer therapy strategy as aberrant DNA methylation is closely related to initiation and progression of many cancers [12]. The link between DNMT1 hyperactivity and cancer is well established [12], [33], making DNMT1 an important cancer drug target. For this reason, we set out to develop a pipeline to discover novel, direct small molecule inhibitors of DNMT1 activity. The *sine qua non* for small molecule screening is a robust assay. We previously refined an *in vitro* assay for DNMT1 that couples DNA methylation to fluorescence generation using the restriction endonuclease GlaI [27], allowing for an activity assay that is exquisitely sensitive. This assay utilizes a hemimethylated hairpin DNA substrate with a 5′-FAM fluorophore and a 3′ quencher. The fully methylated product oligonucleotide is a substrate for the restriction endonuclease GlaI. Cleavage of the product DNA releases the fluorophore from the quencher and generates fluorescence. Using this endonuclease-coupled DNA methylation assay, we showed that an N-terminal deletion of sequences up to and including the RFTS domain, the first 620 amino acids, results in an enzyme that is 640-fold more active [27]. This de-repressed form of DNMT1, with a *k*_cat_/*K*_m_ of ∼10^6^ M^−1^ s^−1^ [27], has sufficient catalytic power to allow for facile identification of inhibitors using the fluorogenic assay.

**Figure 1:**
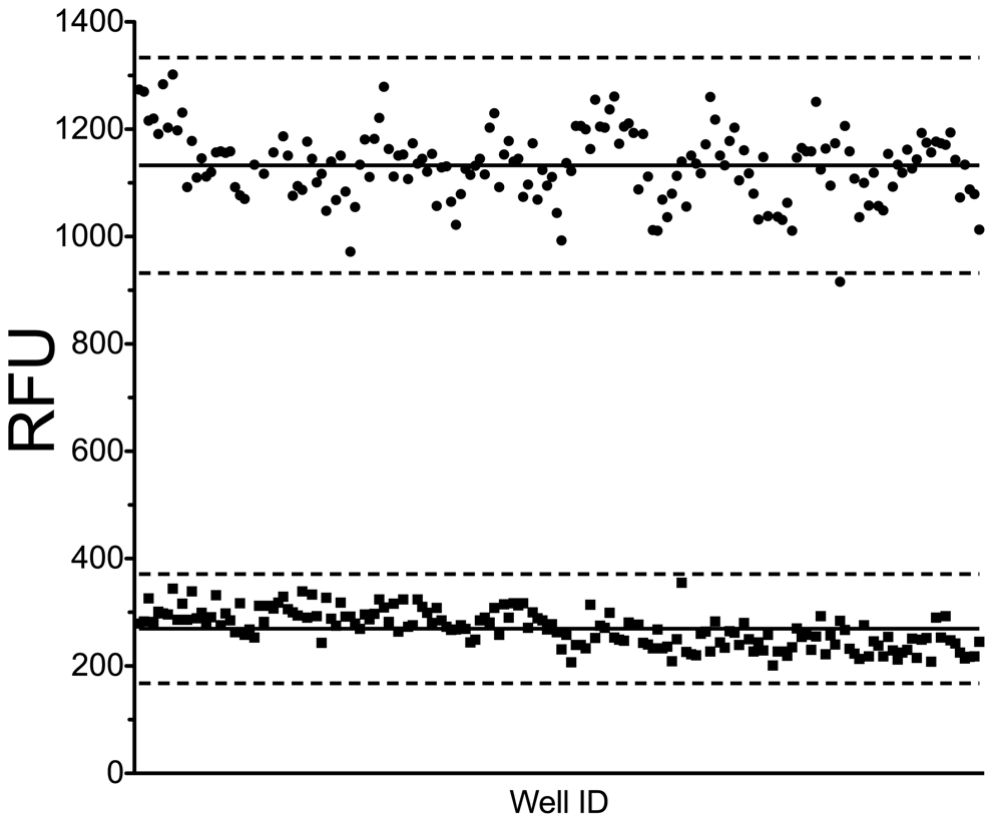
Z’-factor determination of HTS assay in 384 well plates. In a 384 well plate, 192 wells were used as negative controls (•), DMSO in the presence of DNMT1 and GlaI, and 192 wells were used as positive controls (▪), DMSO in the presence of GlaI alone. The solid line represents the mean value of the positive and negative controls, 267 and 1133 respectively. The dashed lines represent 3 standard deviations above and below the averages. The Z’-factor calculated from this data is 0.66.

To determine if the endonuclease-coupled DNA methylation assay is suitable for HTS, we first evaluated the effect of DMSO on the observed activity of the de-repressed form of DNMT1 (amino acids 621–1616). The presence of up to 5% DMSO, the highest value tested, has no effect on DNMT1 activity (Fig. S1), indicating that DMSO does not retard DNMT1 or inhibit the DNA methylation detection system. Next, to increase throughput, we wished to ensure the assay could be miniaturized to 384 well plates. Range-finding experiments at a variety of substrate concentrations, enzyme amounts, volumes and time were performed. Conditions were chosen in which the DNA substrate is at 20 nM, 10–20 times *K*_m,DNA_ [27], [34], to bias against selection of DNA-competitive inhibitors, SAM is at 10 µM, 5 times *K*_m,SAM_ [34], to permit identification of SAM competitors, and reactions were scored 25 minutes post enzyme addition. Using DNMT1 plus GlaI endonuclease to represent 100% DNMT1 activity, and GlaI in the absence of DNMT1 to represent 0% DNMT1 activity, a Z’-factor of 0.66 was obtained for the miniaturized DNA methylation assay (Fig. 1). The S/B ratio (4.2) and S/N ratio (12.9) indicate that the assay is robust for HTS.

**Figure 2:**
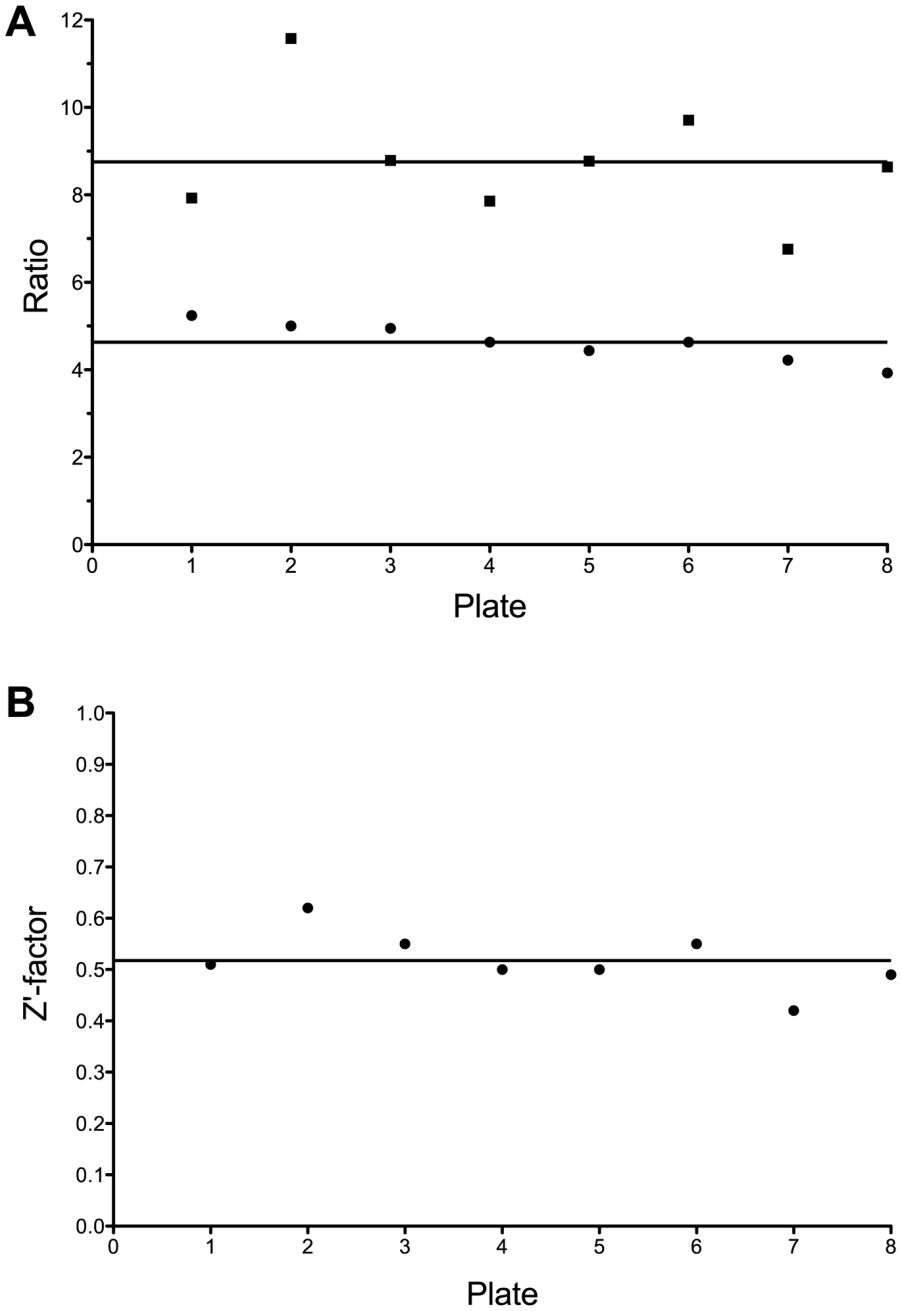
Quality control of Spectrum HTS experiment. A) The S/N ratio (▪) and S/B ratio (•) were calculated from control wells on each of the 8 384 well plates in the screen. Average values across the screen are represented by a solid line. The average S/N ratio was 8.8±1.4. The average S/B ratio was 4.6±0.4. B) The Z’-factor of each plate was calculated using the positive and negative controls on the plate. The average across the screen, represented by a solid line, was 0.52±0.06.

**Figure 3:**
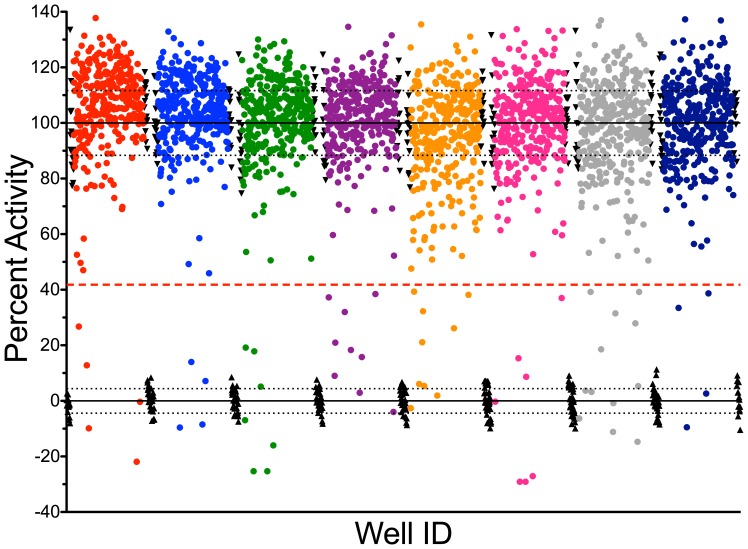
HTS screen of the Spectrum library. Raw fluorescence data from the Spectrum screen were normalized to the assay controls on each plate. The normalized data are shown as circles with each assay plate being a different color. The solid lines represent the average value of the positive (▴) and negative (▾) controls. The dashed black lines represent one standard deviation from the positive and negative controls. The red dashed line represents 5 standard deviations below the average negative control. Compounds below this cutoff were considered primary hits. 57 of the 2320 compounds were identified as hits.

The miniaturized endonuclease-coupled DNA methylation assay was used to screen the Spectrum compound collection for inhibitors of DNMT1 activity. These 2320 compounds provide a wide-range of biological activities and structural diversity. The collection contains drug and drug-like synthetic compounds as well as natural products. The quality of the assay was assessed by determining the S/B ratio, S/N ratio and Z’-factor of each of the eight 384 well plates in the screen (Fig. 2). The average values across the screen are similar to the values obtained in the control assay. With average S/B ratio, S/N ratio and Z’-factor values of 4.6±0.4, 8.8±1.4 and 0.52±0.06 respectively (Fig. 2), screening of the Spectrum collection was successful. Compounds were picked as hits if resulting activities were greater than 5 standard deviations below the mean of the negative controls (Fig. 3). Of 2320 compounds examined, 57 (2.5%) were primary hits. Each of the 57 primary hits was re-tested in triplicate for DNMT1 inhibition using the endonuclease-coupled DNA methylation assay. For validation, time-dependent reactions traces for each candidate inhibitor and a DMSO control reaction were collected. 11 of 57 primary hits failed to inhibit DNMT1 (Table S1), whereas 46 compounds inhibited product formation by at least 40% (Table S1), giving an apparent hit rate of ∼2%.

### Identification of Direct DNMT1 Inhibitors

Direct DNMT1 inhibitors have the potential to be useful molecular probes and possibly leads for drug development. To ensure that the inhibitory effect exhibited by the validated HTS hits stems from a direct interaction with DNMT1, we employed DSF [32] to determine the observed melting temperature (*T_m_*) of DNMT1 in the presence of each candidate inhibitor. Compounds that directly interact with target proteins in the absence of substrates frequently stabilize against thermal denaturation and shift the observed *T_m_* to right. Thus, comparing the *T_m_* observed in the presence of a small molecule to that observed in the presence of DMSO should allow for detection of direct inhibitors. Of 46 compounds tested, five could not be assayed by DSF. These compounds either strongly quenched the Sypro Orange fluorescence signal or were fluorescent and interfered with the assay. The majority of compounds assayed had no significant effect of the observed melting temperature (Table S2). Addition of 27 of the 41 successfully assayed compounds resulted in *T_m_* values within ∼0.5°C of that observed for DNMT1 alone. However, addition of 12 compounds shifted the observed *T_m_* to the right by at least 0.9°C (Table S2), indicating that the compounds make a stabilizing interaction with the protein.

Though 12 compounds bind DNMT1 directly as indicated by DSF, we aimed to eliminate those compounds that interfere with an aspect of the endonuclease-coupled DNA methylation assay, making it difficult to determine their effect on DNMT1 activity. To assess the possibility that the small molecules inhibit GlaI or quench fluorescence of the 5′-FAM product, the 12 candidate DNMT1 inhibitors were counterscreened against GlaI in a fluorogenic assay using a fully CpG-methylated hairpin DNA substrate (Fig. S2). Addition of 10 of the 12 compounds resulted in activities ≥79% of that observed with DMSO alone (Table S3). However, addition of two compounds resulted in observed activities of <40%. Due to their interference with the coupling reaction used in the DNA methylation assay, these compounds were not considered DNMT1 inhibitors.

To further investigate inhibition of DNMT1 by the direct-binding compounds, a detergent test was performed. Inhibition of each compound was investigated in the presence and absence of 0.01% Triton X-100 to eliminate promiscuous molecules that inhibit by nonspecific aggregation of protein targets [35]. The presence of Triton X-100 did not reduce the inhibition observed for 9 compounds (Table S4). However, observed inhibition was almost completely lost in the presence of detergent for one candidate inhibitor. For this reason, this compound was excluded from further study.

**Figure 4:**
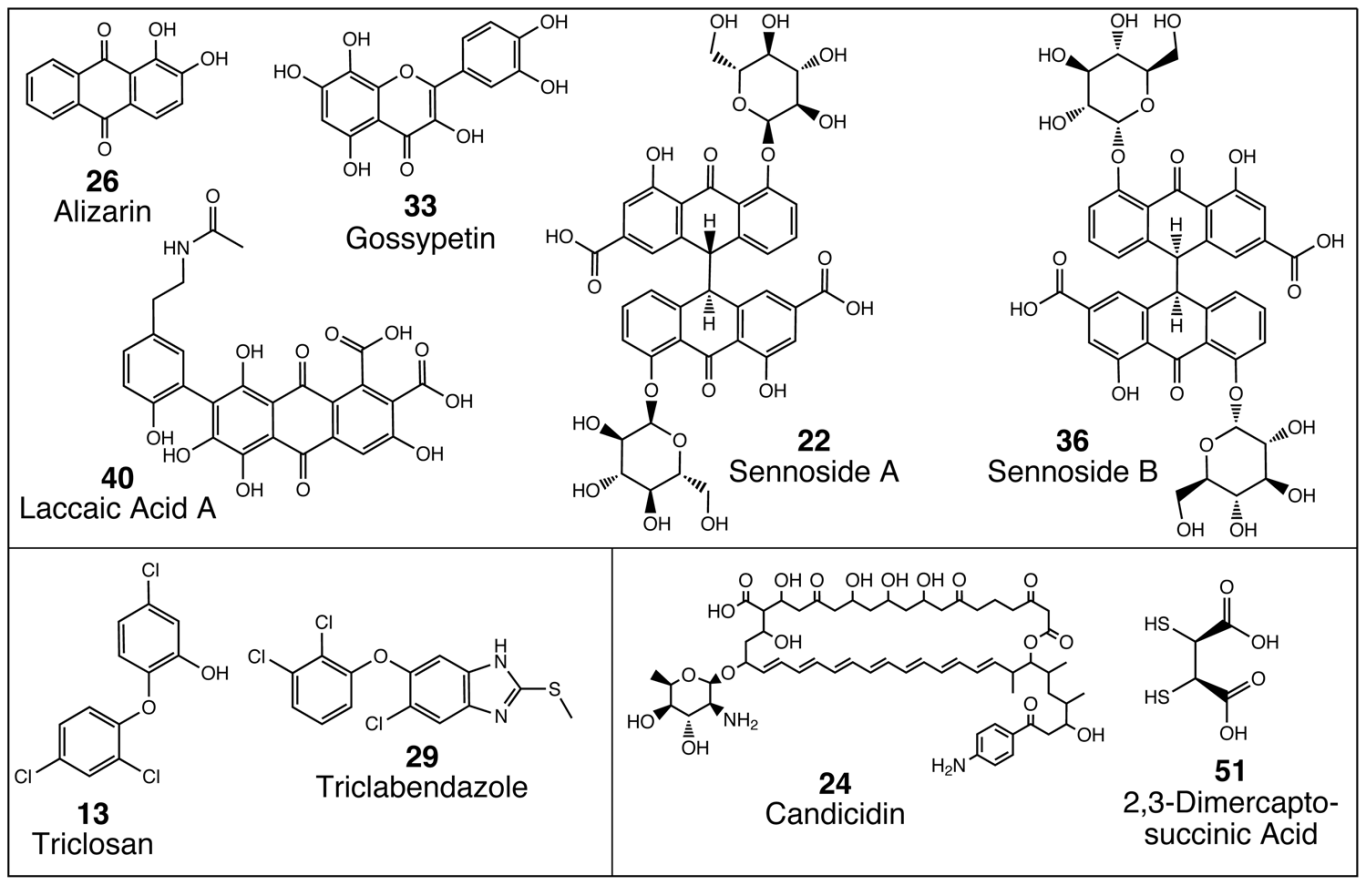
Structure of identified direct DNMT1 inhibitors. Shown are the 9 identified inhibitors from the Spectrum collection. Compound numbers correspond to those in all tables and Figure 5. Five compounds contain a quinone-like substructure. Two identified inhibitors are chlorobenzene compounds.

Finally, while the compounds shift the observed *T_m_* of DNMT1 in the absence of DNA, indicating that they directly interact with the enzyme, we sought to ensure that the mechanisms of inhibition of these compounds are not due to DNA intercalation. To address this possibility, the compounds were added to a DNA-ethidium bromide mixture. If the compounds compete with ethidium bromide and intercalate into DNA, the fluorescence intensity of ethidium bromide will decrease. None of the validated direct hits were capable of decreasing ethidium bromide fluorescence, indicating that they are not strong DNA intercalators (Table S5). In comparison, addition of daunorubicin, a known DNA intercalator [36], significantly reduced fluorescence in this assay. Overall, the high throughput pipeline described resulted in the discovery of 9 direct inhibitors of DNMT1 enzymatic activity (Fig. 4) from 2320 compounds in the Spectrum collection.

### Concentration-Dependence of Inhibition

**Figure 5:**
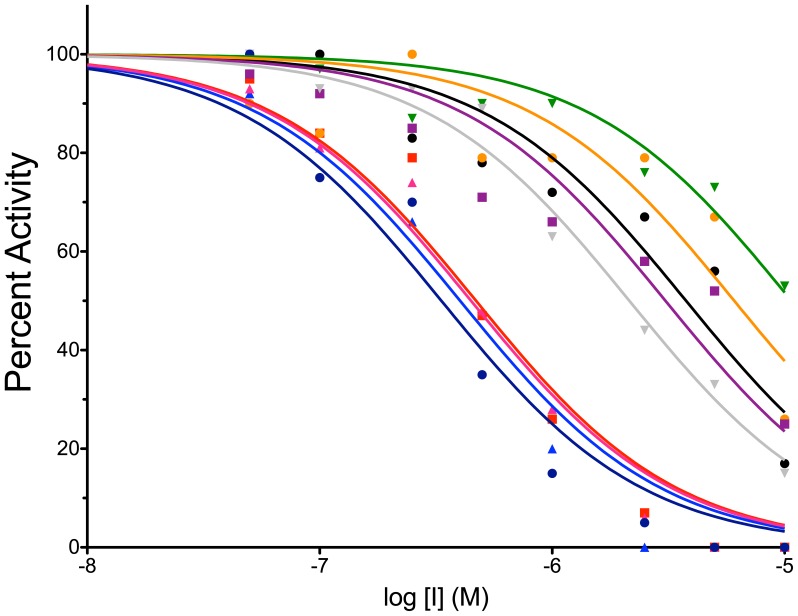
Dose-response analysis of the identified direct DNMT1 inhibitors. IC_50_ values were determined at 20 nM oligonucleotide 8006 and 10 µM SAM for each inhibitor (13– black •; 22– red ▪; 24– blue ▴; 26– green ▾; 29– orange •; 33– purple ▪; 36– pink ▴, 40– grey ▾, 51– navy •). Inhibitor concentration was varied from 0.1–10 µM. The percent activity was determined by comparing to an uninhibited DMSO-containing control. The data were fit using a Hill slope of -1 to obtain IC_50_ values (Table 1).

To gauge the potency of the 9 resulting compounds, the concentration-dependence of inhibition was investigated using the endonuclease-coupled DNA methylation assay. Under identical conditions (10 µM SAM and 20 nM hairpin oligonucleotide 8006), each inhibitor was varied from 100 nM to 10 µM. The percent activity observed at each condition was determined by comparing to an uninhibited DMSO-containing control reaction. The 9 compounds examined exhibited IC_50_ values ranging from 300 nM to 11 µM (Fig. 5 and Table 1).

**Table 1:** IC_50_ values of the 9 compounds identified using the high throughput pipeline.

Cmpd #	Assay Plate	Well ID	Cmpd ID	IC_50_ (µM)*
13	3	F8	01505465	3.8 (2.4–5.9)
22	4	G5	01504078	0.47 (0.33–0.67)
24	4	J5	01503867	0.40 (0.27–0.60)
26	5	B22	00210850	11 (7.9–15)
29	5	M9	01505786	6.1 (3.4–11)
33	6	A4	01505143	2.9 (1.7–5.0)
36	6	G15	01504080	0.45 (0.33–0.60)
40	6	K10	01505847	2.1 (1.7–2.6)
51	7	O19	00201507	0.33 (0.22–0.51)

* IC_50_ values obtained by fitting percent activity data shown in Fig. 5 (with 95% confidence interval).

### Inhibitor Selectivity

Most characterized DNMT inhibitors are not isozyme-selective. With three catalytically active DNMTs in humans and the finding that DNMT3A acts as a tumor suppressor [21], isozyme selectivity of DNMT inhibitors is important. The compounds discovered using this high throughput pipeline are direct inhibitors of the truncated and activated form of DNMT1 (amino acids 621–1616) *in vitro*. To investigate selectivity for inhibition of methylation of oligonucleotide 8006, three methyltransferases was examined: RFTS-containing DNMT1 (amino acids 351–1616), the CD-DNMT3A/DNMT3L complex, and the bacterial methyltransferase from M.SssI. SGI-1027 was used as a positive control because it is has been shown to inhibit each enzyme [25]. 5-aza-cytidine was used as a negative control; this nucleoside inhibitor must be incorporated into DNA to exert inhibitory effects on DNMTs. The percent activity observed for each enzyme was determined by comparing to an uninhibited DMSO-containing control assay. As expected, SGI-1027 inhibited the activity of all DNMTs tested, while addition of 5-aza-cytidine did not inhibit any enzyme examined (Table 2). Seven of nine compounds discovered in this HTS campaign showed some selectivity. Whereas triclosan (cmpd 13) and alizarin (cmpd 26) inhibited each enzyme to a similar degree, the other seven compounds either showed a preference for inhibition of DNMT1 or for inhibition of both DNMT1 and M.SssI, but not DNMT3A/DNMT3L. The observed selectivity for DNMT1 over DNMT3A/DNMT3L in this assay was two to four-fold.

**Table 2:** Percent activity observed using three different DNMTs.

Compound #	RFTS-containing Dnmt1	DNMT3A/DNMT3L Complex	M.SssI DNMT
13	25±8	23±10	25±4
22	40±10	88±11	20±5
24	N.D.*	49±16	49±12
26	51±10	49±12	68±11
29	N.D.	110±5	19±6
33	38±13	74±13	86±9
36	30±12	83±15	40±7
40	21±9	80±10	82±10
51	24±11	61±12	36±7
SGI-1027	N.D.	N.D.	N.D.
5-aza-cytidine	100±11	97±10	100±5

* N.D. – No detectable activity observed above background.

## Discussion

Drastic changes to normal DNA methylation patterns occur in malignancy. The genome typically becomes globally hypomethylated with regional hypermethylation and gene silencing of tumor suppressor genes [12]. Targeting these epigenetic changes is a promising cancer therapy strategy. While modified cytosine nucleosides, such as 5-aza-cytidine, have been approved to treat myelodysplastic syndrome, such demethylators are not DNMT isozyme specific and require incorporation into DNA to exert their effects [22], suggesting that there is room for improvement in probe development. Molecular probes targeted at DNMT1 enzyme activity will also be useful in furthering our understanding of cancer etiology. Such molecules can be used to determine the degree to which DNA methyltransferase enzyme activity as opposed to DNMT physical interactions is important for gene silencing and carcinogenesis. Direct inhibitors may also serve as lead compounds for new cancer therapeutics. Thus, the objective of developing this high throughput pipeline was to identify novel direct inhibitors of DNMT1 activity.

The HTS assay described herein is ultrasensitive and requires only 0.24 pmol of DNMT1 (amino acids 621–1616) per well. In comparison, a recently described scintillation proximity assay [29] required almost 0.9 pmol of a similar DNMT1 construct. In contrast, a different endonuclease-coupled assay that uses biotin to attach a DNA substrate to a microtiter plate [37] used nearly 17 pmol of full-length DNMT1 per reaction. Our fluorescence-based DNA methylation assay for DNMT inhibitors requires little manipulation. The assay can be accomplished by simply adding test compounds and enzyme to assay solution and reading the fluorescence generated after a short 37°C incubation. Less than 1 hour is required from plate set-up to final reading, making the assay simple and scalable.

The initial screen of the Spectrum collection yielded 57 hits that inhibited DNMT1 activity by at least 5 standard deviations below the negative control. Following hit validation, 46 compounds reliably inhibited fluorescence-generation in the endonuclease-coupled DNA methylation assay. The validated inhibitors were examined for their ability to bind directly to DNMT1 using DSF [32]. These assays can be performed in 96 well and 384 well plates in a real-time PCR machine, permitting HTS for direct inhibition. Twelve compounds were determined to bind directly with DNMT1 on account of their ability to shift the observed *T_m_*. A shift of the observed *T_m_* of at least 0.9°C was used as cutoff for direct binders. This is in line with previous studies that have shown changes in *T_m_* of 1–2°C or greater for direct binders of other targets [32], [38], [39]. Of the 12 compounds that stabilize DNMT1 against thermal denaturation, 10 were further confirmed as lacking in GlaI inhibition and 9 were confirmed for their ability to inhibit in the presence of detergent. These secondary and confirmatory assays reduced the number of direct DNMT1 inhibitors to nine, 0.39% of all compounds screened.

All 9 inhibitors discovered using this high throughput pipeline show concentration-dependent inhibition of DNMT1 activity *in vitro*, with IC_50_ values ranging from 0.3–11 µM (Fig. 5 and Table 1). The majority of compounds identified are polycyclic aromatics (Fig. 4). Though many such compounds are DNA intercalators, these molecules did not inhibit GlaI cleavage of the DNMT1 product oligonucleotide, they shifted the melting temperature of DNMT1 in DSF assays that included no DNA, and they failed to compete with ethidium bromide in DNA intercalation assays. Compounds similar in structure to our identified compounds, such as nitroflavones and dichlone [28], were recently reported to inhibit DNMT3A, suggesting that polycyclic aromatics may profitably be screened for inhibition of DNMT isozymes.

Our high throughput pipeline used a truncated form of DNMT1 (amino acids 621–1616) due to its increased *in vitro* activity [27]. To examine the ability of the identified compounds to inhibit full-length DNMT1, we tested them against RFTS-containing DNMT1 (amino acids 351–1616), a protein that behaves similarly to the full-length enzyme *in vitro* [27]. All of the identified compounds inhibited the activity of RFTS-containing DNMT1 (Table 2). To examine isozyme selectivity, the compounds were also screened against the CD-DNMT3A/DNMT3L complex and the bacterial DNMT from M.SssI. Interestingly, only alizarin (cmpd 26) and triclosan (cmpd 13) appear to be nonselective. The seven other compounds identified in the HTS campaign exhibited two to four-fold selectivity for DNMT1 over DNMT3A/DNMT3L. While these initial finding are promising, further work is needed to characterize the new inhibitors. Structure activity relationship analyses of the inhibitors and related compounds could reveal more potent and specific inhibitors.

Of the compounds identified in our screen, five contain anthracene or anthraquinone-related structures. Laccaic acid A (LCA, cmpd 40), a highly substituted anthraquinone natural product, is ∼5-fold more potent than alizarin (cmpd 26), an anthraquinone with only two hydroxyl substituents, suggesting that substituted anthraquinones represent a novel pharmacophore for DNMT1 inhibitors. LCA exhibited ∼4-fold selectivity for DNMT1, while alizarin inhibited all DNMTs examined equally. Further characterization of LCA has shown that it is a DNA-competitive inhibitor, which reactivates expression of a set of methylation-silenced genes in MCF-7 breast cancer cells [34]. In addition, LCA reverses DNMT1-dependent oncogenic transformation and apoptosis in murine *Rgs6−/−* mouse embryonic fibroblasts [40].

This screen also yielded two chlorobenzene compounds, triclosan (cmpd 13) and triclabendazole (cmpd 29), with similar potencies against the activated form of DNMT1. A recent study has shown that treatment with triclosan reduced the levels of DNA methylation in HepG2 cells [41]. Triclabendazole but not triclosan exhibited selectivity for DNMT1 versus DNMT3A/DNMT3L.

The high throughput pipeline described in this study was used successfully to identify direct inhibitors of DNMT activity *in vitro* from a small chemical library. Whereas alizarin and triclosan appear to be nonspecific DNMT inhibitors, seven other compounds appear to be at least partially selective for DNMT1 over DNMT3A/DNMT3L. Ongoing experiments are designed to determine their mechanisms of inhibition, cellular availability and cellular isozyme specificity. Structure activity relationship data and co-crystallization studies are expected to aid in further defining DNMT1 pharmacophores. The pipeline described herein can be used to screen larger and more diverse libraries of chemical matter to discover additional tool compounds and leads for clinical DNMT1 inhibition.

## Supporting Information

Figure S1 **DMSO tolerance of DNMT1.** DNMT1 activity was assayed at 20 nM oligonucleotide 8006 and 10 µM SAM using 2 nM DNMT1. DMSO concentration was varied from 0–5% (0– filled circles; 0.5– filled squares; 1– filled triangles; 2– filled diamonds; 3– open circles; 4– open squares; 5– open triangles). Addition of DMSO has little effect on the observed activity of DNMT1. RFU, relative fluorescence unit.
(TIF)

Figure S2 **GlaI counterscreen.** The effect of each compound on GlaI activity, the coupling enzyme used in the DNA methylation assay, was investigated using an internally quenched hairpin DNA with a fully methylated GCGC site (the cleavage site of GlaI). GlaI cleavage of the oligonucleotide releases the 5′ fluorophore from the 3′ quencher, generating fluorescence in real-time. Shown is the time-dependent cleavage of 5 nM oligonucleotide substrate 8007 with 0.2 U of enzyme in the presence of DMSO (black •) or 11 µM of each compound (13– red •; 22– blue •; 24– green •; 26– purple •; 29– red ▪; 30– blue ▪; 33– green ▪; 36– purple ▪; 40– red ♦; 44– blue ♦; 51– green ♦; 53– purple ♦).
(TIF)

Table S1 **Validation of the initial 57 hits from the Spectrum HTS assay.** Initial hits were validated as DNMT1 inhibitors using the endonuclease-coupled DNA methylation assay. Each compound was assayed in triplicate. Shown is the fluorescence observed following enzyme addition and 25-minute incubation at 37°C. In addition, observed initial velocities were determined from GlaI-corrected, time-dependent reaction traces. The percent activity observed for each inhibitor was determined by comparing to an uninhibited DMSO-containing control reaction. 11 compounds failed to inhibit DNMT1 activity in validation assays.
(DOCX)

Table S2 **Melting temperature of DNMT1 determined using DSF.** DSF was used to determine the observed melting temperature (*T_m_*) of DNMT1 in the presence and absence of validated hits. 12 compounds stabilized DNMT1 against thermal denaturation and shifted the observed *T_m_* to right by at least 0.9°C, indicating that they bind directly to DNMT1.
(DOCX)

Table S3 **Effect of compounds on GlaI endonuclease activity.** A GlaI counterscreen was performed to determine if the compounds inhibit the restriction enzyme used in the DNA methylation assay. Two of the twelve compounds that shifted the melting temperature of DNMT1 inhibited GlaI activity in this assay. These compounds were not studied further.
(DOCX)

Table S4 **Effect of detergent of inhibition.** The percent activity observed using 5 µM compound in the presence and absence of 0.01% Triton X-100 was determined. The observed inhibition with compound 44 was sensitive to detergent. The inhibitory effect of the other nine compounds examined was not sensitive to detergent.
(DOCX)

Table S5 **DNA Intercalation Assay.** DNA intercalation activities of candidate inhibitors were assessed using an assay containing calf thymus DNA and ethidium bromide. Ethidium bromide fluorescence was measured using excitation and emission wavelengths of 320 and 600 nm, respectively. Compounds that intercalate DNA decrease the observed fluorescence. Daunorubicin, a known DNA intercalator, was used as a positive control and significantly reduced the fluorescence signal. None of the compounds identified in the HTS campaign had a significant effect on observed fluorescence, indicating that they do not intercalate into DNA under reaction conditions.
(DOCX)
