# Supplementary figures and images for: An Ultrasensitive High Throughput Screen for DNA Methyltransferase 1-Targeted Molecular Probes
Source: PLoS One. 2013 Nov 13;8(11):e78752. doi: 10.1371/journal.pone.0078752 (PMC3827244; doi:10.1371/journal.pone.0078752)

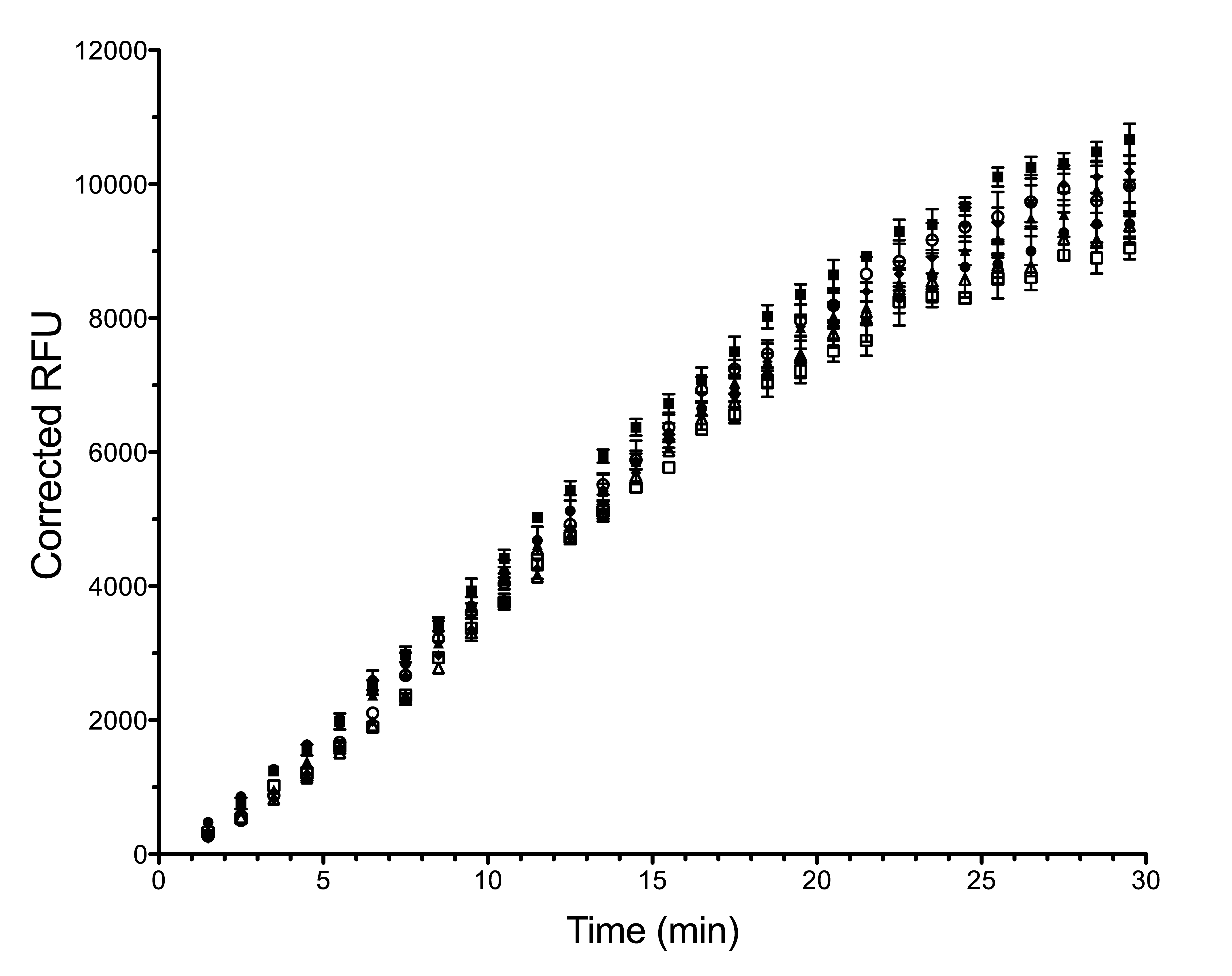

Supplement: Figure S1 — DMSO tolerance of DNMT1. DNMT1 activity was assayed at 20 nM oligonucleotide 8006 and 10 µM SAM using 2 nM DNMT1. DMSO concentration was varied from 0–5% (0– filled circles; 0.5– filled squares; 1– filled triangles; 2– filled diamonds; 3– open circles; 4– open squares; 5– open triangles). Addition of DMSO has little effect on the observed activity of DNMT1. RFU, relative fluorescence unit. (TIF) [file pone.0078752.s001.tif]

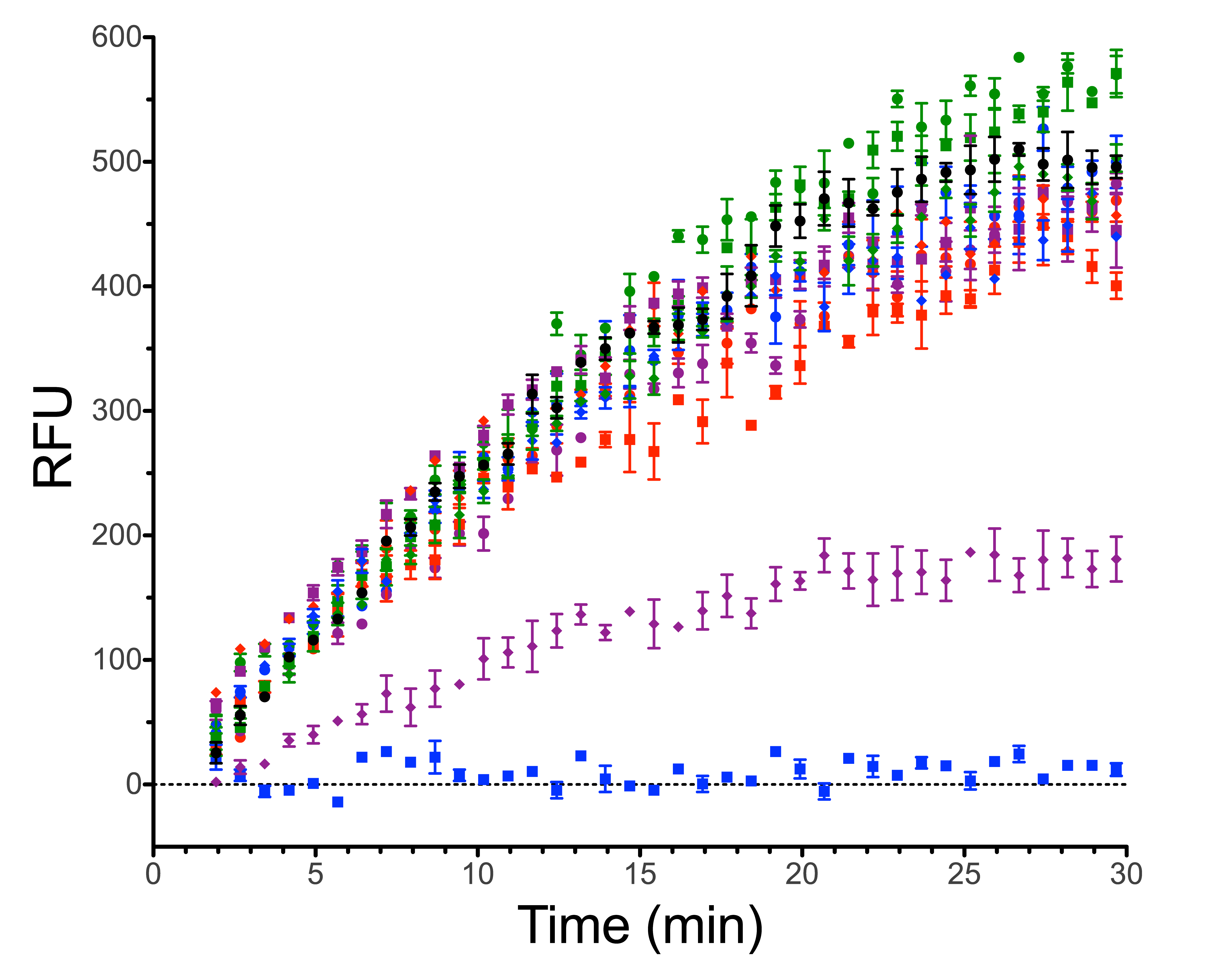

Supplement: Figure S2 — GlaI counterscreen. The effect of each compound on GlaI activity, the coupling enzyme used in the DNA methylation assay, was investigated using an internally quenched hairpin DNA with a fully methylated GCGC site (the cleavage site of GlaI). GlaI cleavage of the oligonucleotide releases the 5′ fluorophore from the 3′ quencher, generating fluorescence in real-time. Shown is the time-dependent cleavage of 5 nM oligonucleotide substrate 8007 with 0.2 U of enzyme in the presence of DMSO (black •) or 11 µM of each compound (13– red •; 22– blue •; 24– green •; 26– purple •; 29– red ▪; 30– blue ▪; 33– green ▪; 36– purple ▪; 40– red ♦; 44– blue ♦; 51– green ♦; 53– purple ♦). (TIF) [file pone.0078752.s002.tif]
