# Supplementary material for: An Ultrasensitive High Throughput Screen for DNA Methyltransferase 1-Targeted Molecular Probes
Source: PLoS One. 2013 Nov 13;8(11):e78752. doi: 10.1371/journal.pone.0078752 (PMC3827244; doi:10.1371/journal.pone.0078752)
Supplement: Table S1 — Validation of the initial 57 hits from the Spectrum HTS assay. Initial hits were validated as DNMT1 inhibitors using the endonuclease-coupled DNA methylation assay. Each compound was assayed in triplicate. Shown is the fluorescence observed following enzyme addition and 25-minute incubation at 37°C. In addition, observed initial velocities were determined from GlaI-corrected, time-dependent reaction traces. The percent activity observed for each inhibitor was determined by comparing to an uninhibited DMSO-containing control reaction. 11 compounds failed to inhibit DNMT1 activity in validation assays. (DOCX) [file pone.0078752.s003.docx]

**Table S1. Validation of the initial 57 hits from the Spectrum HTS assay.**

| **Cmpd #** | **Assay Plate** | **Well ID** | **Cmpd ID** | **MW** | **RFU*** | **Normalized RFU** | ***v_o_***** | **Percent Activity** |
| --- | --- | --- | --- | --- | --- | --- | --- | --- |
|  |  |  |  |  |  |  |  |  |
| DMSO^1^ | - | - | - | - | 1860 ± 151 | 100 | 55 ± 3 | 100 |
| GlaI^2^ | - | - | - | - | 530 ± 48 | 0 | - | - |
| 1 | 1 | A21 | 330001 | 1255.4 | 203 ± 18 | -25 | N.D.^3^ | 0 |
| 2 | 1 | F7 | 01504105 | 1701.2 | 378 ± 7 | -11 | N.D. | 0 |
| 3 | 1 | H4 | 01500119 | 428.8 | 935 ± 19 | 30 | 24 ± 2 | 44 |
| 4 | 1 | M6 | 02300009 | 410.6 | 629 ± 34 | 7 | 17 ± 2 | 31 |
| 5 | 1 | P21 | 01504218 | 388.5 | 526 ± 31 | 0 | 10 ± 2 | 18 |
| 6 | 2 | E9 | 01500210 | 512.3 | **1747 ± 43** | **91** | **52 ± 3** | **95** |
| 7 | 2 | I12 | 01500344 | 76.1 | **1863 ± 189** | **100** | **58 ± 3** | **105** |
| 8 | 2 | J16 | 01500687 | 383.4 | **1710 ± 61** | **89** | **58 ± 2** | **105** |
| 9 | 2 | L15 | 01500549 | 270.3 | **1723 ± 62** | **90** | **51 ± 2** | **93** |
| 10 | 3 | A4 | 01503239 | 356.5 | 450 ± 94 | -6 | 10 ± 2 | 18 |
| 11 | 3 | D10 | 01505444 | 337.9 | 140 ± 23 | -29 | N.D. | 0 |
| 12 | 3 | E6 | 01503278 | 517.4 | 31 ± 6 | -38 | N.D. | 0 |
| 13 | 3 | F8 | 01505465 | 289.5 | 779 ± 42 | 19 | 15 ± 2 | 27 |
| 14 | 3 | H6 | 01505483 | 543.5 | 834 ± 10 | 23 | 12 ± 2 | 22 |
| 15 | 3 | N3 | 01505308 | 682.2 | 732 ± 79 | 15 | N.D. | 0 |
| 16 | 3 | O11 | 01503223 | 675.7 | 647 ± 20 | 9 | 13 ± 2 | 24 |
| 17 | 4 | A10 | 01504417 | 504.5 | 700 ± 16 | 13 | 5 ± 1 | 9 |
| 18 | 4 | A17 | 01505974 | 473.4 | 1291 ± 53 | 57 | 32 ± 2 | 58 |
| 19 | 4 | C13 | 01505782 | 546.6 | 588 ± 52 | 4 | N.D. | 0 |
| 20 | 4 | C22 | 01502032 | 1429.2 | 501 ± 10 | -2 | N.D. | 0 |
| 21 | 4 | E8 | 01502033 | 446.4 | 721 ± 49 | 14 | 15 ± 2 | 27 |
| 22 | 4 | G5 | 01504078 | 862.8 | 621 ± 12 | 7 | N.D. | 0 |
| 23 | 4 | I12 | 01300017 | 960.8 | 661 ± 17 | 10 | 5 ± 1 | 9 |
| 24 | 4 | J5 | 01503867 | 1109.3 | 677 ± 60 | 11 | 5 ± 1 | 9 |
| 25 | 4 | L3 | 01503873 | 591.7 | **1480 ± 19** | **71** | **42 ± 2** | **76** |
| 26 | 5 | B22 | 00210850 | 240.2 | 1024 ± 110 | 37 | 27 ± 2 | 49 |
| 27 | 5 | C3 | 01505168 | 343.4 | 1209 ± 47 | 51 | N.D. | 0 |
| 28 | 5 | C10 | 01502329 | 910.0 | 143 ± 8 | -29 | N.D. | 0 |
| 29 | 5 | M9 | 01505786 | 359.7 | 889 ± 20 | 27 | 20 ± 2 | 36 |
| 30 | 5 | M11 | 01503806 | 394.3 | 217 ± 17 | -24 | N.D. | 0 |
| 31 | 5 | N13 | 01506191 | 305.8 | 254 ± 1 | -21 | 5 ± 1 | 9 |
| 32 | 5 | P11 | 01502245 | 302.2 | 620 ± 34 | 7 | N.D. | 0 |
| 33 | 6 | A4 | 01505143 | 318.2 | 1145 ± 147 | 46 | 25 ± 3 | 45 |
| 34 | 6 | B3 | 01500690 | 232.3 | **1417 ± 59** | **67** | **45 ± 3** | **82** |
| 35 | 6 | F6 | 00100223 | 456.5 | **1646 ± 95** | **84** | **57 ± 2** | **104** |
| 36 | 6 | G15 | 01504080 | 862.8 | 591 ± 41 | 5 | N.D. | 0 |
| 37 | 6 | H5 | 01500861 | 399.9 | 651 ± 52 | 9 | 16 ± 2 | 29 |
| 38 | 6 | I19 | 01504176 | 319.4 | 1118 ± 29 | 44 | 28 ± 2 | 51 |
| 39 | 6 | J6 | 00100584 | 474.6 | **1824 ± 29** | **97** | **58 ± 3** | **105** |
| 40 | 6 | K10 | 01505847 | 537.4 | 602 ± 4 | 5 | 6 ± 1 | 11 |
| 41 | 6 | O6 | 00700024 | 598.7 | 534 ± 6 | 0 | N.D. | 0 |
| 42 | 7 | B3 | 00100013 | 544.6 | **1612 ± 136** | **81** | **56 ± 2** | **102** |
| 43 | 7 | B19 | 01500721 | 254.2 | 632 ± 45 | 8 | 6 ± 2 | 11 |
| 44 | 7 | C20 | 00300038 | 174.2 | 509 ± 25 | -2 | N.D. | 0 |
| 45 | 7 | F6 | 00300554 | 274.3 | **1429 ± 98** | **68** | **40 ± 2** | **73** |
| 46 | 7 | F9 | 01500802 | 142.1 | 866 ± 83 | 25 | 19 ± 2 | 35 |
| 47 | 7 | G13 | 00201182 | 318.2 | 519 ± 94 | -1 | N.D. | 0 |
| 48 | 7 | J6 | 00200090 | 254.3 | **1578 ± 132** | **79** | **43 ± 2** | **78** |
| 49 | 7 | L12 | 01505163 | 422.4 | 408 ± 28 | -9 | 12 ± 1 | 22 |
| 50 | 7 | L19 | 01505876 | 992.8 | 427 ± 27 | -8 | N.D. | 0 |
| 51 | 7 | N12 | 01505007 | 182.2 | 612 ± 55 | 6 | 5 ± 1 | 9 |
| 52 | 7 | O4 | 01500759 | 272.2 | 471 ± 12 | -4 | 6 ± 1 | 11 |
| 53 | 7 | O19 | 00201507 | 914.7 | 574 ± 41 | 3 | N.D. | 0 |
| 54 | 8 | E15 | 01505164 | 498.5 | 440 ± 41 | -7 | N.D. | 0 |
| 55 | 8 | G7 | 01505331 | 246.3 | 944 ± 16 | 31 | 18 ± 2 | 33 |
| 56 | 8 | M9 | 01504820 | 808.9 | 504 ± 91 | -2 | N.D. | 0 |
| 57 | 8 | O15 | 01505272 | 230.3 | 933 ± 132 | 30 | 15 ± 1 | 27 |

*RFU: Relative fluorescence unit observed after 25-minute incubation at 37 ºC. Average ± standard deviation of three replicates. Bolded values are within 3 standard deviation of the uninhibited control.

**Initial velocity (RFU/min) calculated from the first 12 minutes of GlaI corrected triplicate reaction traces. Bolded values are less than 30% inhibited.

^1^DMSO containing control reaction. Represent 100% activity.

^2^GlaI control reaction in the absence of DNMT1. Represent 0% activity.

^3^N.D. – No detectable activity was observed
