# Supplementary material for: An Ultrasensitive High Throughput Screen for DNA Methyltransferase 1-Targeted Molecular Probes
Source: PLoS One. 2013 Nov 13;8(11):e78752. doi: 10.1371/journal.pone.0078752 (PMC3827244; doi:10.1371/journal.pone.0078752)
Supplement: Table S2 — Melting temperature of DNMT1 determined using DSF. DSF was used to determine the observed melting temperature (Tm) of DNMT1 in the presence and absence of validated hits. 12 compounds stabilized DNMT1 against thermal denaturation and shifted the observed Tm to right by at least 0.9°C, indicating that they bind directly to DNMT1. (DOCX) [file pone.0078752.s004.docx]

**Table S2. Melting temperature of DNMT1 determined using DSF.**

| **Cmpd #** | **Assay Plate** | **Well ID** | **Cmpd ID** | **MW** | ***T_m_* (ºC)** | **∆*T_m_*** |
| --- | --- | --- | --- | --- | --- | --- |
|  |  |  |  |  |  |  |
| DMSO | - | - | - | - | 46.5 ± 0.1 | - |
| 1 | 1 | A21 | 330001 | 1255.4 | 46.7 ± 0.1 | +0.2 |
| 2 | 1 | F7 | 01504105 | 1701.2 | N.D.* | - |
| 3 | 1 | H4 | 01500119 | 428.8 | 46.8 ± 0.1 | +0.3 |
| 4 | 1 | M6 | 02300009 | 410.6 | 46.4 ± 0.1 | -0.1 |
| 5 | 1 | P21 | 01504218 | 388.5 | 46.8 ± 0.1 | +0.3 |
| 10 | 3 | A4 | 01503239 | 356.5 | 46.3 ± 0.1 | -0.2 |
| 11 | 3 | D10 | 01505444 | 337.9 | 44.8 ± 0.3 | -1.7 |
| 12 | 3 | E6 | 01503278 | 517.4 | 47.1 ± 0.1 | +0.6 |
| 13 | 3 | F8 | 01505465 | 289.5 | 47.5 ± 0.1 | +1.0 |
| 14 | 3 | H6 | 01505483 | 543.5 | 46.5 ± 0.1 | 0 |
| 15 | 3 | N3 | 01505308 | 682.2 | N.D. | - |
| 16 | 3 | O11 | 01503223 | 675.7 | 46.9 ±0.1 | +0.4 |
| 17 | 4 | A10 | 01504417 | 504.5 | 46.7 ± 0.1 | +0.2 |
| 18 | 4 | A17 | 01505974 | 473.4 | 46.8 ± 0.1 | +0.3 |
| 19 | 4 | C13 | 01505782 | 546.6 | 46.9 ± 0.1 | +0.4 |
| 20 | 4 | C22 | 01502032 | 1429.2 | 46.0 ± 0.1 | -0.5 |
| 21 | 4 | E8 | 01502033 | 446.4 | 46.8 ± 0.1 | +0.3 |
| 22 | 4 | G5 | 01504078 | 862.8 | 47.5 ± 0.1 | +1.0 |
| 23 | 4 | I12 | 01300017 | 960.8 | N.D. | - |
| 24 | 4 | J5 | 01503867 | 1109.3 | 47.5 ± 0.1 | +1.0 |
| 26 | 5 | B22 | 00210850 | 240.2 | 48.5 ± 0.1 | +2.0 |
| 27 | 5 | C3 | 01505168 | 343.4 | 46.2 ± 0.1 | -0.3 |
| 28 | 5 | C10 | 01502329 | 910.0 | 46.6 ± 0.1 | +0.1 |
| 29 | 5 | M9 | 01505786 | 359.7 | 48.3 ± 0.1 | +1.8 |
| 30 | 5 | M11 | 01503806 | 394.3 | 48.6 ± 0.1 | +2.1 |
| 31 | 5 | N13 | 01506191 | 305.8 | 47.1 ± 0.1 | +0.5 |
| 32 | 5 | P11 | 01502245 | 302.2 | 46.5 ± 0.1 | 0 |
| 33 | 6 | A4 | 01505143 | 318.2 | 64.4 ± 0.2 | +17.9 |
| 36 | 6 | G15 | 01504080 | 862.8 | 47.4 ± 0.1 | +0.9 |
| 37 | 6 | H5 | 01500861 | 399.9 | 46.4 ± 0.1 | -0.1 |
| 38 | 6 | I19 | 01504176 | 319.4 | 46.8 ± 0.1 | +0.3 |
| 40 | 6 | K10 | 01505847 | 537.4 | 48.5 ± 0.1 | +2.0 |
| 41 | 6 | O6 | 00700024 | 598.7 | N.D. | - |
| 43 | 7 | B19 | 01500721 | 254.2 | 46.3 ± 0.1 | -0.2 |
| 44 | 7 | C20 | 00300038 | 174.2 | 47.4 ± 0.1 | +0.9 |
| 46 | 7 | F9 | 01500802 | 142.1 | 46.1 ± 0.1 | -0.4 |
| 47 | 7 | G13 | 00201182 | 318.2 | 45.8 ± 0.1 | -0.7 |
| 49 | 7 | L12 | 01505163 | 422.4 | 45.8 ± 0.2 | -0.7 |
| 50 | 7 | L19 | 01505876 | 992.8 | N.D. | - |
| 51 | 7 | N12 | 01505007 | 182.2 | 49.0 ± 0.1 | +2.5 |
| 52 | 7 | O4 | 01500759 | 272.2 | 46.4 ± 0.3 | -0.1 |
| 53 | 7 | O19 | 00201507 | 914.7 | 54.9 ± 0.1 | +8.4 |
| 54 | 8 | E15 | 01505164 | 498.5 | 45.4 ± 0.1 | -1.1 |
| 55 | 8 | G7 | 01505331 | 246.3 | 46.7 ± 0.1 | +0.2 |
| 56 | 8 | M9 | 01504820 | 808.9 | 45.8 ± 0.1 | -0.7 |
| 57 | 8 | O15 | 01505272 | 230.3 | 46.4 ± 0.1 | -0.1 |

*N.D. – *T_m_* not determined due to interfering intrinsic compound fluorescence or compound quenching Sypro Orange fluorescence.
