# Supplementary material for: An Ultrasensitive High Throughput Screen for DNA Methyltransferase 1-Targeted Molecular Probes
Source: PLoS One. 2013 Nov 13;8(11):e78752. doi: 10.1371/journal.pone.0078752 (PMC3827244; doi:10.1371/journal.pone.0078752)
Supplement: Table S3 — Effect of compounds on GlaI endonuclease activity. A GlaI counterscreen was performed to determine if the compounds inhibit the restriction enzyme used in the DNA methylation assay. Two of the twelve compounds that shifted the melting temperature of DNMT1 inhibited GlaI activity in this assay. These compounds were not studied further. (DOCX) [file pone.0078752.s005.docx]

**Table S3. Effect of compounds on GlaI endonuclease activity.**

| Cmpd # | Assay Plate | Well ID | Cmpd ID | Initial Velocity (RFU/min) | Percent Activity |
| --- | --- | --- | --- | --- | --- |
| 13 | 3 | F8 | 01505465 | 28 ± 2 | 104 |
| 22 | 4 | G5 | 01504078 | 24 ± 1 | 86 |
| 24 | 4 | J5 | 01503867 | 27 ± 1 | 96 |
| 26 | 5 | B22 | 00210850 | 22 ± 1 | 79 |
| 29 | 5 | M9 | 01505786 | 22 ± 2 | 79 |
| 30 | 5 | M11 | 01503806 | N.D.* | 0 |
| 33 | 6 | A4 | 01505143 | 27 ± 2 | 96 |
| 36 | 6 | G15 | 01504080 | 28 ± 1 | 104 |
| 40 | 6 | K10 | 01505847 | 22 ± 3 | 79 |
| 44 | 7 | C20 | 00300038 | 25 ± 1 | 89 |
| 51 | 7 | N12 | 01505007 | 25 ± 1 | 89 |
| 53 | 7 | O19 | 00201507 | 11 ± 1 | 39 |
| DMSO | - | - | - | 27 ± 1 | 100 |

*N.D. – No detectable activity was observed.
