# Supplementary material for: An Ultrasensitive High Throughput Screen for DNA Methyltransferase 1-Targeted Molecular Probes
Source: PLoS One. 2013 Nov 13;8(11):e78752. doi: 10.1371/journal.pone.0078752 (PMC3827244; doi:10.1371/journal.pone.0078752)
Supplement: Table S4 — Effect of detergent of inhibition. The percent activity observed using 5 µM compound in the presence and absence of 0.01% Triton X-100 was determined. The observed inhibition with compound 44 was sensitive to detergent. The inhibitory effect of the other nine compounds examined was not sensitive to detergent. (DOCX) [file pone.0078752.s006.docx]

**Table S4. Effect of detergent of inhibition.**

|  |  |  |  | 0.01% Triton X-100 | |
| --- | --- | --- | --- | --- | --- |
| Cmpd # | Assay Plate | Well ID | Cmpd ID | - | + |
| 13 | 3 | F8 | 01505465 | 52 | 48 |
| 22 | 4 | G5 | 01504078 | 7 | 9 |
| 24 | 4 | J5 | 01503867 | 0 | 0 |
| 26 | 5 | B22 | 00210850 | 67 | 63 |
| 29 | 5 | M9 | 01505786 | 52 | 46 |
| 33 | 6 | A4 | 01505143 | 53 | 47 |
| 36 | 6 | G15 | 01504080 | 0 | 0 |
| 40 | 6 | K10 | 01505847 | 56 | 58 |
| 44 | 7 | C20 | 00300038 | 8 | 70 |
| 51 | 7 | O19 | 00201507 | 10 | 8 |
| DMSO | - | - | - | 100 | 100 |
