# Supplementary material for: An Ultrasensitive High Throughput Screen for DNA Methyltransferase 1-Targeted Molecular Probes
Source: PLoS One. 2013 Nov 13;8(11):e78752. doi: 10.1371/journal.pone.0078752 (PMC3827244; doi:10.1371/journal.pone.0078752)
Supplement: Table S5 — DNA Intercalation Assay. DNA intercalation activities of candidate inhibitors were assessed using an assay containing calf thymus DNA and ethidium bromide. Ethidium bromide fluorescence was measured using excitation and emission wavelengths of 320 and 600 nm, respectively. Compounds that intercalate DNA decrease the observed fluorescence. Daunorubicin, a known DNA intercalator, was used as a positive control and significantly reduced the fluorescence signal. None of the compounds identified in the HTS campaign had a significant effect on observed fluorescence, indicating that they do not intercalate into DNA under reaction conditions. (DOCX) [file pone.0078752.s007.docx]

**Table S5. DNA Intercalation Assay.**

| Cmpd # | Assay Plate | Well ID | Cmpd ID | RFU* |
| --- | --- | --- | --- | --- |
| 13 | 3 | F8 | 01505465 | 2406 ± 5 |
| 22 | 4 | G5 | 01504078 | 2415 ± 150 |
| 24 | 4 | J5 | 01503867 | 2389 ± 64 |
| 26 | 5 | B22 | 00210850 | 2364 ± 30 |
| 29 | 5 | M9 | 01505786 | 2500 ± 144 |
| 33 | 6 | A4 | 01505143 | 2524 ± 45 |
| 36 | 6 | G15 | 01504080 | 2309 ± 60 |
| 40 | 6 | K10 | 01505847 | 2311 ± 148 |
| 51 | 7 | O19 | 00201507 | 2525 ± 200 |
| Daunorubicin | - | - | - | 605 ± 33 |
| DMSO | - | - | - | 2370 ± 190 |

*RFU: relative fluorescence unit (average ± standard deviation of triplicate assays)
